# Comparative Effectiveness and Safety of Etelcalcetide and Cinacalcet in Hemodialysis Patients with Secondary Hyperparathyroidism: A Multicenter Retrospective Cohort Study

**DOI:** 10.3390/jcm15218271

**Published:** 2026-10-23

**Authors:** Engin Onan, Emrah Günay, Derya Basak Tanburoglu, Ilter Bozaci, Mehmet Horoz, Ruya Kozanoglu, Hasan Micozkadioglu, Refik Olmaz, Ramazan Danis, Kutlay Tutucu, Dilek Torun

**Affiliations:** 1 Department of Nephrology, Baskent University Adana Dr. Turgut Noyan Training and Research Hospital, 01250 Adana, Türkiye; rusancak@hotmail.com (R.K.); hasanmicoz@hotmail.com (H.M.); dilektorun@hotmail.com (D.T.); 2 Department of Nephrology, Adana City Training and Research Hospital, 01230 Adana, Türkiye; egnay01@gmail.com (E.G.); basakkas@hotmail.com (D.B.T.); mehmethoroz@yahoo.com (M.H.); 3 Department of Nephrology, Antalya City Training and Research Hospital, 07080 Antalya, Türkiye; ilterbozaci@gmail.com; 4 Department of Nephrology, Mersin City Training and Research Hospital, 33240 Mersin, Türkiye; rolmaz.1417@gmail.com; 5 Department of Nephrology, Diyarbakir Gazi Yasargil Training and Research Hospital, 21070 Diyarbakir, Türkiye; dr.r.danis@gmail.com; 6 Department of Nephrology, Toros Devlet Hospital, 33050 Mersin, Türkiye; kntutucu@yahoo.com

**Keywords:** etelcalcetide, cinacalcet, hemodialysis, secondary hyperparathyroidism, hypocalcemia, adverse effects

## Abstract

**Background:** Etelcalcetide and cinacalcet are widely used for the treatment of secondary hyperparathyroidism (sHPT) in patients receiving maintenance hemodialysis. This study compared their real-world biochemical effectiveness and adverse-event profiles over 12 months. **Methods:** This multicenter retrospective cohort study included 116 hemodialysis patients with sHPT treated at five dialysis centers. Sixty-three patients received intravenous etelcalcetide and 53 received oral cinacalcet. The primary outcome was the between-group difference in the percentage change in parathyroid hormone (PTH) from baseline to 12 months. Secondary outcomes included longitudinal changes in corrected calcium, phosphorus, albumin, and C-reactive protein levels and the incidence of treatment-related adverse events. **Results:** Baseline PTH levels were higher in the etelcalcetide group than in the cinacalcet group (1562 vs. 1268 pg/mL, *p* = 0.011). PTH levels decreased during the first 3 months and subsequently fluctuated in both groups. The mean 12-month percentage change in PTH was −17.52% in the cinacalcet group and −14.13% in the etelcalcetide group (between-group mean difference, 3.39 percentage points; 95% CI, −20.36 to 27.14; *p* = 0.776). Hypocalcemia occurred more frequently with etelcalcetide than with cinacalcet (52% vs. 32%, *p* = 0.028). In contrast, nausea (68% vs. 2%, *p* = 0.001) and vomiting (8% vs. 0%, *p* = 0.026) were more frequent with cinacalcet. **Conclusions:** No statistically significant difference in the 12-month percentage change in PTH was observed between etelcalcetide and cinacalcet, although baseline PTH levels were higher in the etelcalcetide group. The treatments demonstrated distinct adverse-event profiles, with more frequent hypocalcemia during etelcalcetide treatment and more frequent gastrointestinal adverse events during cinacalcet treatment. These findings describe biochemical outcomes and adverse-event frequencies among patients who remained on treatment for at least 3 months; they do not establish therapeutic equivalence or comparative safety.

## 1. Introduction

Secondary hyperparathyroidism (sHPT) is a common and challenging complication among patients undergoing maintenance hemodialysis [1]. It arises from chronic disturbances in mineral metabolism primarily driven by impaired renal phosphate excretion, reduced calcitriol synthesis, and persistent parathyroid gland stimulation. Uncontrolled sHPT is associated with significant morbidity including vascular calcification, bone disease, and increased cardiovascular risk, making effective management crucial for improving patient outcomes [2].

Beyond PTH, calcium, and phosphate, several interconnected regulators contribute to CKD–mineral and bone disorder. Fibroblast growth factor 23 (FGF-23), a hormone produced predominantly by osteocytes, regulates phosphate handling and vitamin D metabolism [3]. Osteoprotegerin (OPG) acts as a decoy receptor for RANKL, thereby modulating osteoclast differentiation and bone resorption [4]. Sclerostin, another osteocyte-derived protein, inhibits Wnt/β-catenin signaling and osteoblast-mediated bone formation [5]. Together, these mediators illustrate the complex regulation of mineral metabolism and bone remodeling in CKD.

Calcimimetics, such as cinacalcet and etelcalcetide, play a pivotal role in sHPT treatment by enhancing the sensitivity of parathyroid cells to calcium-sensing receptors, leading to a reduction in parathyroid hormone (PTH) levels [6,7]. Cinacalcet, an oral agent, has been widely used for over a decade. Etelcalcetide, an intravenous calcimimetic, is an alternative that may improve adherence and mitigate gastrointestinal side effects [8]. Despite their established efficacy, comparative data on their long-term effects, safety profiles, and impact on key biochemical markers remain limited.

This study aimed to evaluate the real-world effectiveness and safety of etelcalcetide and cinacalcet in patients undergoing hemodialysis with sHPT. By analyzing changes in PTH levels, calcium, phosphorus, inflammatory markers, and adverse events over a 12-month period, we sought to provide insights into their relative benefits and risks. Understanding these differences is essential for optimizing individualized treatment strategies and improving clinical outcomes in this vulnerable patient population.

## 2. Materials and Methods

### 2.1. Study Design and Patient Selection

This retrospective cohort study was conducted across five dialysis centers. Hemodialysis was performed using biocompatible membranes. This study included hemodialysis patients treated with either intravenous etelcalcetide or oral cinacalcet for secondary hyperparathyroidism. The inclusion criteria were as follows: (1) at least 6 months of regular hemodialysis treatment, (2) baseline PTH ≥ 600 pg/mL, and (3) at least 3 months of continuous treatment with either drug. Patients with severe cardiovascular disease, active malignancy, or discontinuation of the study treatment because of severe adverse effects were excluded. Thus, patients who discontinued treatment before completing 3 months were not included. Because a complete screening log was unavailable, the number of patients who discontinued treatment early could not be ascertained retrospectively. Patients received either cinacalcet or etelcalcetide for secondary hyperparathyroidism management. Cinacalcet was administered orally, starting at 30 mg/day, and titrated according to PTH response, serum calcium and phosphate levels, and tolerability. In this cohort, the highest dose used and tolerated in routine clinical practice was 90 mg/day; no patient received a dose above 90 mg/day. Etelcalcetide was administered intravenously after hemodialysis sessions, starting at 5 mg three times per week, with a dose titration of up to 15 mg three times per week, depending on biochemical responses. Dose modifications were made according to routine clinical practice to ensure optimal PTH suppression, while maintaining serum calcium levels within the target range. As this was a retrospective cohort study, all eligible patients meeting inclusion criteria during the study period were included without prior sample size calculation. Of 150 patients screened for eligibility, 116 met the inclusion criteria and were included in the final analysis. The remaining 34 patients were excluded due to insufficient treatment duration, missing data, or comorbid conditions. During the 12-month follow-up of the 116 included patients, no deaths, kidney transplants, transfers to another center, or treatment changes were recorded.

Baseline concomitant treatments for CKD–mineral and bone disorder, including calcitriol and paricalcitol use, as well as dialysis adequacy parameters, were recorded.

### 2.2. Data Collection and Outcomes

Baseline demographic data, including age, sex, dialysis duration, weight, and comorbidities, were recorded. Dialysis access type was also recorded. Residual kidney function was not systematically assessed or recorded in the study dataset and was therefore unavailable for analysis. Prior cinacalcet use before etelcalcetide initiation was recorded when available. Biochemical parameters, including PTH, calcium, phosphorus, albumin, and C-reactive protein (CRP) levels, were assessed at baseline and at 3, 6, 9, and 12 months post-treatment. All patients were followed for a total of 12 months. The average follow-up time was 12 months per patient, totaling 1392 person-months. Hemoglobin values were available in routine clinical records but were not collected as a continuous study variable. Anemia-related events were recorded as a binary outcome (present/absent) when a hemoglobin value < 10 g/dL was documented during treatment. Erythropoiesis-stimulating agents were administered as clinically indicated; however, ESA initiation and dose-adjustment strategies were not systematically recorded. Adverse events were retrospectively identified from routine clinical records and laboratory data. No study-specific standardized adverse-event surveillance protocol was used across the participating centers; therefore, events were captured as documented in routine clinical practice. Serum calcium was corrected for albumin using the following formula: corrected calcium (mg/dL) = measured total calcium (mg/dL) + 0.8 × [4 − serum albumin (g/dL)]. For the study analyses, calcium values were evaluated at baseline and at months 3, 6, 9, and 12. Hypocalcemia was defined as a corrected serum calcium level < 8 mg/dL at any post-baseline assessment. Symptoms, severity grading, and treatment consequences were not systematically recorded; therefore, the safety analysis reflects biochemically documented hypocalcemia. The primary endpoint was the percentage change in PTH levels, whereas the secondary endpoints included changes in calcium, phosphorus, C-reactive protein (CRP), and adverse event incidence. The percentage change in PTH was calculated for each patient as [(PTH at follow-up − baseline PTH)/baseline PTH] × 100; therefore, negative values indicate a reduction in PTH. This study was conducted in accordance with the Declaration of Helsinki and was approved by the Baskent University Medical and Health Sciences Research Board (Project No. KA24/465; approval date: 18 March 2025). Informed consent was waived because of the retrospective design and use of anonymized data.

### 2.3. Statistical Analysis

Descriptive statistics are presented as mean ± standard deviation (SD) or median with interquartile range (IQR) for continuous variables and as percentages for categorical variables. Comparisons between treatment groups were conducted using independent sample *t*-tests or Mann–Whitney U tests for continuous data, and chi-square or Fisher’s exact tests for categorical variables. Repeated measures analysis of variance (ANOVA) was used to evaluate changes in biochemical parameters over time. Multiple linear regression was used to assess the association between treatment group and 12-month PTH level, adjusting for baseline PTH, body weight, and dialysis duration. Multivariable logistic regression was used to assess factors associated with hypocalcemia during follow-up, including treatment group, baseline corrected calcium, calcium-based phosphate binder use, and dialysis duration. Post-baseline calcium measurements were not included as predictors in the hypocalcemia model. Statistical significance was set at *p* < 0.05. For within-group comparisons of non-normally distributed variables over time, the Wilcoxon signed-rank test was used as appropriate.

## 3. Results

A total of 116 hemodialysis patients were included in the study, of whom 61 (52.6%) were female and 55 (47.4%) were male. Of the 63 patients receiving etelcalcetide, 20 (31.7%) had received cinacalcet during the preceding three months, whereas 43 (68.3%) were cinacalcet-naïve. Decisions to switch from cinacalcet to etelcalcetide were made by the treating clinicians due to inadequate response or intolerance; however, the specific reason for switching was not consistently documented for each patient and could not be reliably classified retrospectively. The mean age was 48.39 ± 14.06 years, and the mean dialysis duration was 103.58 ± 76.39 months. Most patients (83.6%) had an arteriovenous fistula for dialysis access, whereas 16.4% used a catheter. Baseline calcitriol use was comparable between the cinacalcet and etelcalcetide groups (66.0% vs. 58.7%, *p* = 0.419), as was paricalcitol use (11.3% vs. 23.8%, *p* = 0.082). The overall use of any active vitamin D analog was also similar between the groups (77.4% vs. 77.8%, *p* = 0.957). Baseline dialysis adequacy parameters, including Kt/V and URR, did not differ significantly between the groups (Table 1).

Baseline PTH levels were significantly higher in the etelcalcetide group than in the cinacalcet group (1562 vs. 1268 pg/mL, *p* = 0.011) (Table 1). No statistically significant between-group differences in PTH levels were observed at 3, 6, 9, or 12 months (*p* = 0.054, *p* = 0.188, *p* = 0.056, and *p* = 0.404, respectively). At 12 months, the mean percentage change in PTH was −17.52% ± 42.63% in the cinacalcet group (*n* = 30) and −14.13% ± 41.43% in the etelcalcetide group (*n* = 22). The between-group mean difference (etelcalcetide minus cinacalcet) was 3.39 percentage points (95% CI, −20.36 to 27.14; *p* = 0.776) (Table 2).

In the subgroup analysis according to prior cinacalcet use, baseline PTH differed across the cinacalcet, etelcalcetide without recent cinacalcet use, and etelcalcetide after recent cinacalcet use groups (median, 1268, 1493, and 1633 pg/mL, respectively; overall *p* = 0.030); however, none of the Bonferroni-adjusted pairwise comparisons were statistically significant. Baseline CRP was higher in the etelcalcetide group with recent prior cinacalcet use than in the etelcalcetide group without recent cinacalcet use (5.8 vs. 2.0 mg/L; adjusted *p* = 0.004). At 12 months, the median percentage change in PTH was −15.6% (*n* = 30), −12.0% (*n* = 10), and −21.0% (*n* = 12), respectively, with no significant difference across the three groups (overall *p* = 0.894). PTH percentage change at the last available visit also did not differ significantly (overall *p* = 0.550). The proportions of patients achieving a ≥30% reduction in PTH did not differ across groups at 12 months or at the last available visit (overall *p* = 1.000 and *p* = 0.579, respectively; Supplementary Tables S3 and S4).

Serum calcium levels decreased significantly from baseline to month 3 in the etelcalcetide group (*p* = 0.004), with no significant changes at subsequent time points. In the cinacalcet group, calcium levels increased modestly at month 9 compared with baseline (*p* = 0.026). Hypocalcemia (<8 mg/dL) occurred more frequently in the etelcalcetide group than in the cinacalcet group (52% vs. 32%, *p* = 0.028) (Table 3). Detailed values for calcium, phosphorus, albumin, and CRP at each time point are presented in Supplementary Table S1. CRP levels decreased significantly from baseline to month 9 in the cinacalcet group (*p* = 0.004), whereas no significant within-group changes were observed in the etelcalcetide group. The remaining within-group comparisons were not statistically significant. Detailed pairwise comparisons of CRP levels are presented in Supplementary Table S2.

Adverse event analysis demonstrated that nausea and vomiting were significantly more common in the cinacalcet group (nausea, 68% vs. 2%, *p* = 0.001; vomiting, 8% vs. 0%, *p* = 0.026), whereas hypocalcemia was more prevalent in the etelcalcetide group (52% vs. 32%, *p* = 0.028) (Table 3). There were no significant differences in the incidences of hypotension, headache, anemia, or hypertension between the groups.

Figure 1 illustrates the longitudinal changes in mean PTH and corrected calcium levels and the incidence of key adverse events in both treatment groups. PTH levels decreased during the first 3 months and subsequently fluctuated in both groups; however, the percentage change in PTH from baseline to 12 months did not differ significantly between the groups (*p* = 0.776). Corrected calcium levels showed modest fluctuations throughout follow-up. Hypocalcemia was more frequent with etelcalcetide, whereas nausea and vomiting were more frequent with cinacalcet.

In the multivariable linear regression model, 12-month PTH did not differ significantly between the etelcalcetide and cinacalcet groups after adjustment for baseline PTH, body weight, and dialysis duration (adjusted coefficient, −70.7 pg/mL; 95% CI, −480.0 to 338.5; *p* = 0.730; *n* = 51) (Table 4). In the multivariable logistic regression model, etelcalcetide treatment was not significantly associated with hypocalcemia during follow-up after adjustment for baseline corrected calcium, calcium-based phosphate binder use, and dialysis duration (adjusted OR, 1.28; 95% CI, 0.51–3.25; *p* = 0.598; *n* = 116) (Table 5). Baseline characteristics and PTH responses according to prior cinacalcet use are presented in Supplementary Tables S3 and S4.

## 4. Discussion

In this multicenter retrospective cohort of 116 hemodialysis patients with secondary hyperparathyroidism, both treatment groups showed an initial reduction in PTH levels followed by fluctuations during subsequent follow-up. Despite higher baseline PTH levels in the etelcalcetide group, no statistically significant between-group difference was observed in the percentage change in PTH from baseline to 12 months. The treatments demonstrated distinct adverse-event profiles: hypocalcemia was more frequent with etelcalcetide, whereas nausea and vomiting were more frequent with cinacalcet.

Secondary hyperparathyroidism remains a major clinical challenge in patients receiving maintenance hemodialysis because of its association with mineral and bone disorders, vascular calcification, and adverse cardiovascular outcomes [9]. Etelcalcetide and cinacalcet lower PTH by enhancing the sensitivity of the calcium-sensing receptor but differ in their routes of administration and adverse-effect profiles [10]. In the 26-week randomized trial by Block et al., etelcalcetide met the noninferiority criterion for achieving a >30% reduction in mean predialysis PTH concentrations and also showed statistically significant superiority for this endpoint (68.2% vs. 57.7%; *p* = 0.004). The difference in study design, endpoint, and follow-up duration limits direct comparison with our 12-month analysis of individual percentage change in PTH [11]. In the US-DOPPS analysis by Karaboyas et al., better PTH control was observed in facilities using etelcalcetide as the primary calcimimetic and in facilities that switched from cinacalcet-first to etelcalcetide-first practice. However, this was a facility-level observational analysis and residual confounding remained possible. Our study adds patient-level data from a five-center cohort with 12-month follow-up and an analysis according to prior cinacalcet use. We found no statistically significant difference in 12-month PTH percentage change between the groups. Separately, in the adjusted linear regression model of 12-month PTH level, treatment group was not significantly associated with PTH after adjustment for baseline PTH, body weight, and dialysis duration (adjusted coefficient for etelcalcetide vs. cinacalcet, −70.7 pg/mL; 95% CI, −480.0 to 338.5; *p* = 0.730). This model included 51 patients, and its wide confidence interval indicates limited precision; these findings should not be interpreted as evidence of therapeutic equivalence.

The clearest distinction between the two treatments was observed in their adverse-event profiles. Hypocalcemia was more frequent with etelcalcetide, consistent with previous reports. Pereira et al. similarly reported an increased incidence of hypocalcemia following etelcalcetide treatment, although most cases were asymptomatic [12]. In contrast, nausea and vomiting were more frequent with cinacalcet, in keeping with its established gastrointestinal adverse-effect profile.

CRP decreased significantly at month 9 in the cinacalcet group, whereas no significant change was observed in the etelcalcetide group. However, this isolated finding should be interpreted cautiously because CRP is subject to substantial biological variability in hemodialysis patients, and the study was not designed to evaluate anti-inflammatory effects.

Oral cinacalcet may present adherence challenges in hemodialysis patients, who are frequently exposed to a high pill burden. The intravenous administration of etelcalcetide may reduce concerns related to daily oral adherence; however, adherence to cinacalcet could not be objectively assessed in this retrospective study, and its contribution to the observed PTH trajectories remains uncertain. Previous studies have associated poor cinacalcet adherence with increased hospitalization and healthcare costs [13]. Route of administration, pill burden, patient preferences, and known adverse-effect profiles may be relevant to clinical decision-making. However, because this study excluded patients who discontinued treatment before completing 3 months, its adverse-event findings should be interpreted descriptively and should not be used to infer the comparative safety of the two treatments.

The increase in PTH levels after the initial 3-month reduction may have been influenced by disease severity, treatment titration, treatment interruptions, hypocalcemia-related dose limitations, concomitant CKD–MBD therapies, or biological variability. Because detailed longitudinal dosing data were unavailable, the present study cannot determine the mechanisms underlying this pattern.

Despite treatment, PTH levels remained markedly elevated in both groups at 12 months, indicating persistent biochemical disease burden. However, the study did not evaluate indications for or subsequent performance of parathyroidectomy.

This study has several important limitations. First, its retrospective and nonrandomized design introduced the potential for treatment-selection bias and residual confounding. Although the adjusted treatment model included treatment group, baseline PTH, body weight, and dialysis duration, residual confounding may remain because other potential confounders, including phosphate-binder use, were not included. Patient-level center identifiers were unavailable; therefore, center effects could not be included in the regression models, and residual confounding related to between-center differences cannot be excluded. Although baseline calcitriol and paricalcitol use was compared between the groups, detailed longitudinal data on dose modifications of active vitamin D analogs and other concomitant CKD–MBD treatments were unavailable. Second, the relatively small sample size may have limited the ability to detect modest between-group differences and precluded reliable assessment of clinical outcomes such as cardiovascular events and mortality. Third, adherence to oral cinacalcet could not be objectively assessed, and the study did not include patient-reported outcomes or quality-of-life measures. The maximum cinacalcet dose used in this cohort was 90 mg/day. Because higher doses were not used, the observed response may not reflect the efficacy achievable with titration to higher doses, up to 180 mg/day. Fourth, detailed longitudinal data on etelcalcetide dose escalation, dose reduction, and temporary treatment interruption were not systematically available. Therefore, we could not determine whether the increase in PTH levels during later follow-up reflected disease progression, insufficient dose titration, dose limitation due to hypocalcemia, or other treatment-related factors. This precludes definitive conclusions regarding a loss of pharmacological response to etelcalcetide. Fifth, patients who discontinued treatment before completing 3 months, including those who discontinued because of severe adverse effects, were not included. The number of such patients could not be determined retrospectively. Therefore, adverse events leading to early discontinuation may not have been captured, and the reported adverse-event proportions only describe patients who remained on treatment for at least 3 months. In addition, because adverse events were identified retrospectively from routine records without a standardized baseline symptom assessment, we could not reliably confirm that every reported symptom was newly incident after treatment initiation. These data cannot establish the comparative safety of the treatments from treatment initiation. Prior cinacalcet treatment and clinician-directed switching because of inadequate response or intolerance may have introduced confounding by indication and may partly explain the higher baseline PTH in the etelcalcetide group. Although we performed subgroup analyses according to prior cinacalcet exposure, the specific reason for switching was not consistently documented for each patient; therefore, residual confounding by indication cannot be excluded. Sixth, the assessment of anemia was limited because hemoglobin was not collected as a continuous study variable, ESA initiation and dose changes were not systematically recorded, and no standardized study-specific anemia-monitoring scale was used. ESA management may have influenced hemoglobin levels during follow-up; therefore, the recorded anemia events should be interpreted cautiously. Finally, although the multicenter design may improve the applicability of the findings to similar hemodialysis populations, differences in healthcare systems, treatment availability, and patient characteristics may limit their broader generalizability.

Future prospective, randomized studies with larger cohorts, standardized dose-titration protocols, systematic assessment of concomitant CKD–MBD therapies, and longer follow-up are needed to confirm these findings and determine whether the treatments differ in patient-centered and clinical outcomes beyond biochemical measures.

## 5. Conclusions

In this multicenter retrospective cohort of hemodialysis patients with secondary hyperparathyroidism, no statistically significant between-group difference was observed in the 12-month percentage change in PTH. Among patients who remained on treatment for at least 3 months, hypocalcemia was recorded more frequently in the etelcalcetide group, whereas nausea and vomiting were recorded more frequently in the cinacalcet group. These findings provide real-world data on the biochemical response and observed adverse-event profiles of the two treatments; the adverse-event findings should be interpreted within the study’s retrospective design and cohort selection.

## Figures and Tables

**Figure 1:**
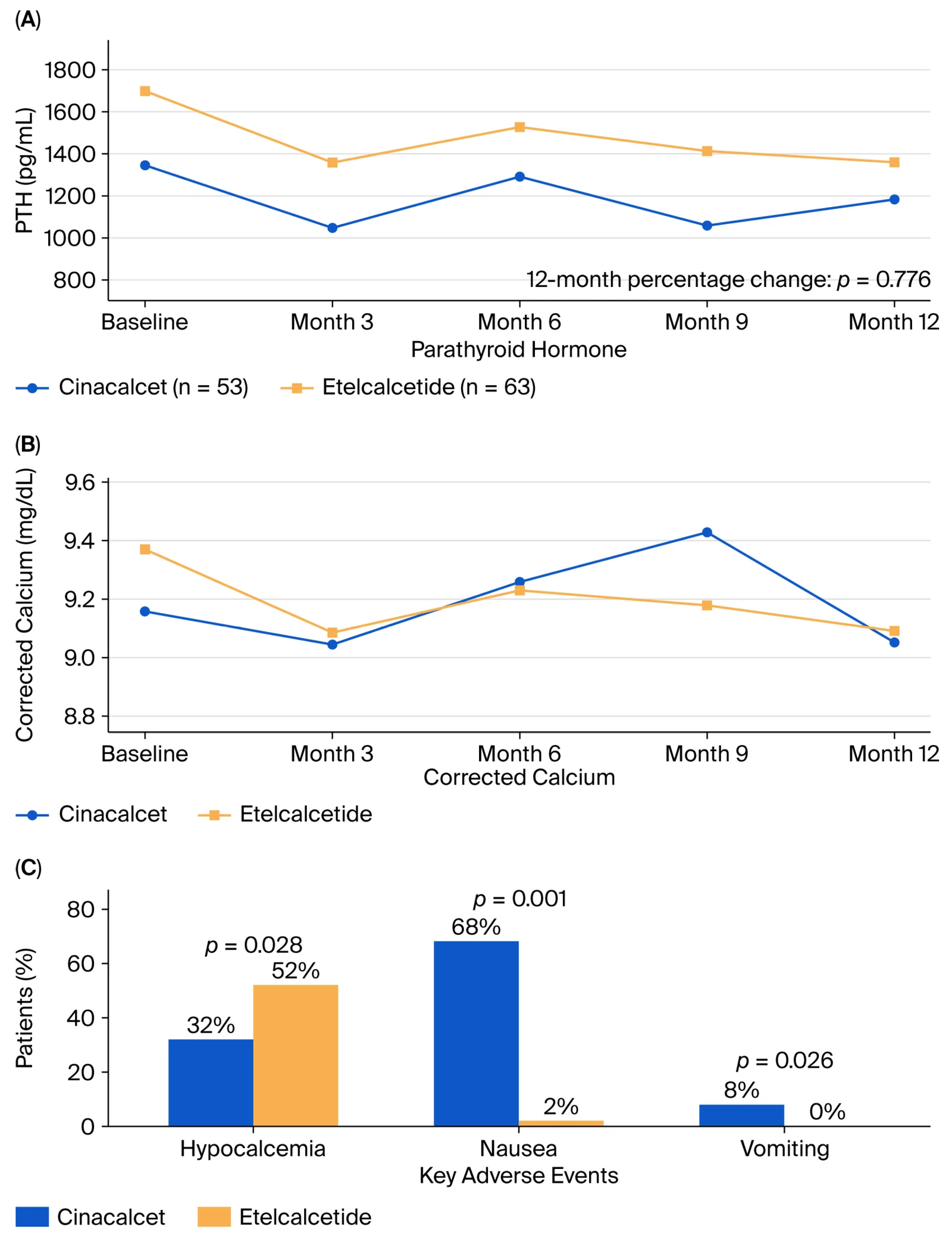
Longitudinal changes in parathyroid hormone and corrected calcium levels and the incidence of key adverse events during 12 months of treatment. Panels (**A**,**B**) show group means. Panel (**C**) shows the proportion of patients with each adverse event.

**Table 1:** Baseline Characteristics of the Study Population.

Variable	Cinacalcet (*n* = 53)	Etelcalcetide (*n* = 63)	*p*-Value
Age (years, mean ± SD)	50.19 ± 13.08	46.87 ± 14.76	0.207
Weight (kg, mean ± SD)	69.22 ± 14.61	60.80 ± 16.03	0.004
Hemodialysis duration (months, median)	111	69	0.001 *
Gender (Female, %)	25 (47.2%)	36 (57.1%)	0.284
Baseline PTH (pg/mL)	1268	1562	0.011 *
Baseline ALP (U/L)	160	167	0.430 *
Baseline Adjusted Calcium (mg/dL)	9.16 ± 0.46	9.37 ± 0.71	0.065
Baseline Phosphorus (mg/dL)	6.02 ± 1.30	5.77 ± 1.21	0.294
Baseline Albumin (g/L)	39.94 ± 3.08	39.17 ± 3.64	0.228
Baseline CRP (mg/L)	3	3.9	0.732 *
Calcitriol use, *n* (%)	35 (66.0%)	37 (58.7%)	0.419
Paricalcitol use, *n* (%)	6 (11.3%)	15 (23.8%)	0.082
Any active vitamin D analog use, *n* (%)	41 (77.4%)	49 (77.8%)	0.957
Baseline Kt/V, median (IQR)	1.52 (1.07–2.10)	1.50 (1.00–3.00)	0.886 *
Baseline URR, median (IQR), %	72 (60–84)	72 (60–91)	0.734 *

Normally distributed continuous variables were compared using the independent-samples *t*-test, non-normally distributed continuous variables were compared using the Mann–Whitney U test, and categorical variables were compared using Pearson’s chi-square test. * Mann–Whitney U test.

**Table 2:** PTH Levels at Follow-Up Visits.

Time Point	Cinacalcet (Mean ± SD)	Etelcalcetide (Mean ± SD)	*p*-Value
3rd Month PTH (pg/mL)	1049 ± 563.53 *n*: 53	1362 ± 1055.97 *n*: 57	0.054
6th Month PTH (pg/mL)	1293 ± 598.68 *n*: 45	1529 ± 973.83 *n*: 40	0.188
9th Month PTH (pg/mL)	1058 ± 652.92 *n*: 39	1413 ± 896.29 *n*: 33	0.056
12th Month PTH (pg/mL)	1185 ± 698.78 *n*: 30	1360 ± 793.36 *n*: 22	0.404
12-month percentage change in PTH from baseline, %, mean ± SD	−17.52 ± 42.63	−14.13 ± 41.43	0.776

**Table 3:** Incidence of Adverse Events.

Adverse Event	Cinacalcet (*n*, %)	Etelcalcetide (*n*, %)	*p*-Value
Hypocalcemia	17 (32%)	33 (52%)	0.028
Nausea	36 (68%)	1 (2%)	0.001
Vomiting	4 (8%)	0 (0%)	0.026
Hypotension	2 (4%)	9 (14%)	0.054
Muscle cramps	2 (4%)	4 (6%)	0.533
Headache	1 (2%)	4 (6%)	0.238
Anemia	7 (13%)	12 (19%)	0.397
Hypertension	0 (0%)	4 (6%)	0.083

**Table 4:** Multivariable Linear Regression Analysis of Factors Associated with 12-Month PTH Level.

Variable	B	Standard Error	Standardized β	t	*p*-Value	95% CI for B
Treatment group (etelcalcetide vs. cinacalcet)	−70.721	203.304	−0.047	−0.348	0.730	−479.951 to 338.509
Baseline PTH (pg/mL)	0.531	0.125	0.522	4.242	<0.001	0.279 to 0.783
Body weight (kg)	−4.949	6.429	−0.101	−0.770	0.445	−17.891 to 7.992
Dialysis duration (months)	−1.598	1.054	−0.199	−1.516	0.136	−3.720 to 0.524
Constant	992.313	543.203	—	1.827	0.074	−101.098 to 2085.725

Model *n* = 51. R = 0.565; R^2^ = 0.320; adjusted R^2^ = 0.260; F = 5.401; *p* = 0.001. B, unstandardized regression coefficient; β, standardized regression coefficient; CI, confidence interval. The cinacalcet group was the reference category. One patient was excluded because body-weight data were unavailable.

**Table 5:** Multivariable Logistic Regression Analysis of Factors Associated with Hypocalcemia During Follow-Up.

Variable	B	Standard Error	Wald	*p*-Value	Adjusted OR	95% CI for OR
Treatment group (etelcalcetide vs. cinacalcet)	0.250	0.474	0.278	0.598	1.28	0.51–3.25
Baseline-corrected calcium (mg/dL)	−0.169	0.353	0.227	0.633	0.84	0.42–1.69
Calcium-based phosphate binder use	1.163	0.498	5.458	0.019	3.20	1.21–8.49
Dialysis duration (months)	−0.005	0.004	2.094	0.148	0.99	0.99–1.00
Constant	1.334	3.195	0.174	0.676	3.80	—

Proposed model: *n* = 116, including 50 hypocalcemia events. Model −2 log likelihood = 144.265; model χ^2^ = 14.331, *p* = 0.006; Nagelkerke R^2^ = 0.156. Hypocalcemia was defined as corrected serum calcium < 8 mg/dL at any post-baseline assessment. OR, odds ratio; CI, confidence interval. The cinacalcet group was the reference category.

## Data Availability

Data supporting the findings of this study are available from the corresponding author upon reasonable request.

## Supplementary Materials

The following supporting information can be downloaded at: https://www.mdpi.com/article/10.3390/jcm15218271/s1, Supplementary Table S1: Biochemical Parameters over Time; Supplementary Table S2: Changes in CRP over Timel; Supplementary Table S3: Baseline characteristics according to recent prior cinacalcet use; Supplementary Table S4: PTH responses according to recent prior cinacalcet use.

## Abbreviations

sHPT	Secondary hyperparathyroidism
PTH	Parathyroid hormone
CRP	C-reactive protein
SD	Standard deviation
IQR	Interquartile range
ANOVA	Analysis of variance
LVH	Left ventricular hypertrophy
FGF23	Fibroblast growth factor 23
NF-κB	Nuclear factor kappa-light-chain-enhancer of activated B cells
TNF-α	Tumor necrosis factor-alpha
IL-1β	Interleukin-1 beta
IL-6	Interleukin-6
PKCδ	Protein kinase C delta
ERK	Extracellular signal-regulated kinase
IRF7	Interferon regulatory factor 7
CKD	Chronic kidney disease
