# Supplementary material for: Comparative Effectiveness and Safety of Etelcalcetide and Cinacalcet in Hemodialysis Patients with Secondary Hyperparathyroidism: A Multicenter Retrospective Cohort Study
Source: J Clin Med. 2026 Oct 23;15(21):8271. doi: 10.3390/jcm15218271 (PMC13764617; doi:10.3390/jcm15218271)
Supplement: Supplementary file 1 [file jcm-15-08271-s001.zip › jcm-4610590-supplementary/Supplementary Table S1_Biochemical Parameters Over Time (2).pdf]

Supplementary Table S1. Biochemical Parameters Over Time

Time Point	Parameter	Cinacalcet	Etelcalcetide	p-value
Baseline	Corrected Calcium	9.16±0,46	9.36±0.71	0.065
Baseline	Phosphorus	6.02±1.30	5.77±1.21	0.294
Baseline	Albumin	40.0 (37 ; 46.5)	39.0 (42 ; 47)	0.194*
Baseline	CRP	3.0 (2 ; 7.5)	3.9 (0.3 ; 96)	0.732*
3rd Month	Corrected Calcium	9.05±0.69	9.08±0.76	0.777
3rd Month	Phosphorus	5.87±1.47	5.44±1.20	0.101
3rd Month	Albumin	40.0 (33.7 ; 48)	39.0 (31 ; 45.3)	0.053*
3rd Month	CRP	3.0 (2 ; 22)	3.45 (0.1 ; 46)	0.915*
6th Month	Corrected Calcium	9.26±0.63	9.23±0.77	0.863
6th Month	Phosphorus	5.90±1.56	5.35±1.34	0.085
6th Month	Albumin	40.2 (31 ; 45)	39.45 (30 ; 47)	0.739*
6th Month	CRP	3.9 (2 ; 156)	3.75 (0,3 ; 258)	0.723*
9th Month	Corrected Calcium	9.43±0.82	9.18±0.91	0.220
9th Month	Phosphorus	5.70±1.43	5.52±1.31	0.578
9th Month	Albumin	39.8 (32 ; 47)	40.0 (22 ; 46)	0.825*
9th Month	CRP	3.3 (2 ; 152)	4.95 (0,3 ; 67)	0.893*
12th Month	Corrected Calcium	9.05±0.89	9.10±0.68	0.865
12th Month	Phosphorus	5.98±1.46	5.85±1,.62	0.773
12th Month	Albumin	40.8 (33 ; 47)	39.5 (34 ; 44)	0.180*
12th Month	CRP	2.0 (2 ; 58,6)	5.7 (1.1 ; 172)	0.059*

For parameters with a normal distribution, means were compared using Student t test, whereas for parameters without a homogeneous distribution, medians were compared using the Mann-Whitney u test . *Marked parameters indicate inhomogeneous distributions. Data are presented as mean ± standard deviation or median (min, max).

Abbreviations: CRP: C reactive protein,
