# Supplementary material for: Comparative Effectiveness and Safety of Etelcalcetide and Cinacalcet in Hemodialysis Patients with Secondary Hyperparathyroidism: A Multicenter Retrospective Cohort Study
Source: J Clin Med. 2026 Oct 23;15(21):8271. doi: 10.3390/jcm15218271 (PMC13764617; doi:10.3390/jcm15218271)
Supplement: Supplementary file 1 [file jcm-15-08271-s001.zip › jcm-4610590-supplementary/Supplementary Table S2_Changes in CRP Over Time (1).pdf]

Supplementary Table S2. Changes in CRP Over Time

Comparison of CRP	Median Change (Cinacalcet)	p-value	Median Change (Etelcalcetide)	p-value
Baseline – 3rd month	0 (3 – 3)	0.715	0.10 (3.90 – 3.45)	0.538
Baseline – 6th month	0 (3 – 3.9)	0.101	0.30 (3.90 – 3.75)	0.744
Baseline – 9th month	0 (3 – 3.3)	**0.004**	0 (3.90 – 4.95)	0.918
Baseline – 12th month	0 (3 – 2)	0.896	0.10 (3.90 – 5.70)	0.578

Values are presented as median (IQR). Wilcoxon signed-rank test was used due to non-normal distribution of CRP values.

Abbreviations: CRP: C reactive protein
