# Supplementary material for: Comparative Effectiveness and Safety of Etelcalcetide and Cinacalcet in Hemodialysis Patients with Secondary Hyperparathyroidism: A Multicenter Retrospective Cohort Study
Source: J Clin Med. 2026 Oct 23;15(21):8271. doi: 10.3390/jcm15218271 (PMC13764617; doi:10.3390/jcm15218271)
Supplement: Supplementary file 1 [file jcm-15-08271-s001.zip › jcm-4610590-supplementary/Supplementary Table S3. Baseline characteristics according to recent prior cinacalcet use.pdf]

Supplementary Table S3. Baseline characteristics according to recent prior cinacalcet use

Variable	Cinacalcet (n = 53)	Etelcalcetide without recent cinacalcet (n = 43)	Etelcalcetide after recent cinacalcet (n = 20)	Overall p	Cinacalcet vs. naïve adj. p	Cinacalcet vs. switch adj. p	Naïve vs. switch adj. p
Age, years	50.2 ± 13.1	46.7 ± 14.5	47.4 ± 15.6	0.445	0.441	0.723	0.982
Female sex, n (%)	25 (47.2)	25 (58.1)	11 (55.0)	0.548	0.854	1.000	1.000
Weight, kg	69.2 ± 14.6	60.1 ± 16.4	62.2 ± 15.5	0.015	0.014	0.202	0.872
Hemodialysis duration, months	111 (72–156)	60 (48–87)	90 (72–114)	<0.001	<0.001	1.000	0.021
Baseline PTH, pg/mL	1268 (1064–1531)	1493 (1158–1990)	1633 (1279–2013)	0.030	0.136	0.061	1.000
Baseline ALP, U/L	160 (117–210)	183 (129–252)	143 (121–193)	0.415	0.735	1.000	0.862
Baseline corrected calcium, mg/dL	9.10 (8.80–9.50)	9.30 (8.90–9.50)	9.30 (8.88–10.43)	0.280	1.000	0.371	1.000
Baseline phosphorus, mg/dL	6.02 ± 1.30	5.82 ± 1.13	5.67 ± 1.39	0.527	0.719	0.548	0.904
Baseline albumin, g/L	40.0 (37.9–41.9)	39.0 (37.2–42.0)	39.0 (37.8–40.5)	0.510	0.950	1.000	1.000
Baseline CRP, mg/L	3.0 (2.0–6.1)	2.0 (1.0–7.0)	5.8 (3.4–18.2)	0.006	0.298	0.135	0.004
Active vitamin D analog use, n (%)	41 (77.4)	33 (76.7)	16 (80.0)	1.000	1.000	1.000	1.000
Study treatment duration, months	12 (8–12)	6 (3–7)	10 (6–16)	<0.001	<0.001	1.000	0.003

Values are presented as mean ± standard deviation, median (minimum–maximum), or n (%), as appropriate. Pairwise p values are Bonferroni-adjusted. Recent prior cinacalcet use was defined as use during the three months before etelcalcetide initiation. ALP, alkaline phosphatase; CRP, C-reactive protein; PTH, parathyroid hormone.
