# Supplementary material for: Comparative Effectiveness and Safety of Etelcalcetide and Cinacalcet in Hemodialysis Patients with Secondary Hyperparathyroidism: A Multicenter Retrospective Cohort Study
Source: J Clin Med. 2026 Oct 23;15(21):8271. doi: 10.3390/jcm15218271 (PMC13764617; doi:10.3390/jcm15218271)
Supplement: Supplementary file 1 [file jcm-15-08271-s001.zip › jcm-4610590-supplementary/Supplementary Table S4. PTH responses according to recent prior cinacalcet use.pdf]

Supplementary Table S4. PTH responses according to recent prior cinacalcet use

Outcome	Cinacalcet (n = 53)	Etelcalcetide without recent cinacalcet (n = 43)	Etelcalcetide after recent cinacalcet (n = 20)	Overall p	Cinacalcet vs. naïve adj. p	Cinacalcet vs. switch adj. p	Naïve vs. switch adj. p
Patients with 12-month PTH value, n	30	10	12	—	—	—	—
12-month PTH change from baseline, %	−15.6 (−50.2 to 5.7)	−12.0 (−44.9 to 15.7)	−21.0 (−37.7 to −6.5)	0.894	1.000	1.000	1.000
≥30% PTH reduction at 12 months, n (%)	11 (36.7)	4 (40.0)	4 (33.3)	1.000	1.000	1.000	1.000
Patients with ≥1 follow-up PTH value, n	53	38	19	—	—	—	—
PTH change at last available visit, %	−8.8 (−46.6 to 10.3)	−12.1 (−44.9 to 5.2)	−8.7 (−26.8 to 11.9)	0.550	1.000	1.000	1.000
≥30% PTH reduction at last available visit, n (%)	17 (32.1)	13 (34.2)	4 (21.1)	0.579	1.000	1.000	0.918

Continuous PTH changes are presented as median (minimum–maximum); categorical outcomes are n (%). The denominators for percentage outcomes are the patients with the corresponding PTH measurement available. Pairwise p values are Bonferroni-adjusted. PTH, parathyroid hormone.
